# Estimate of Impact on the Oral Health-Related Quality of Life of Older Thai People by the Provision of Dentures through the Royal Project

**DOI:** 10.1155/2016/1976013

**Published:** 2016-07-26

**Authors:** Patcharawan Srisilapanan, Narumanas Korwanich, Sutha Jienmaneechotchai, Supranee Dalodom, Nontalee Veerachai, Warangkana Vejvitee, Jeffrey Roseman

**Affiliations:** ^1^Center of Excellence in Dental Public Health, Faculty of Dentistry, Chiang Mai University, Chiang Mai 50200, Thailand; ^2^Bureau of Dental Health, Ministry of Public Health, Nonthaburi 11000, Thailand; ^3^Department of Epidemiology, UAB School of Public Health, University of Alabama, Birmingham, AL 35294-0022, USA

## Abstract

*Purpose.* To estimate the impact of the provision of dentures to Thai older people by the Royal Project on their oral health-related quality of life.* Methods.* A purposive cross-sectional study of a sample of 812 subjects was conducted. The Oral Impacts on Daily Performances (OIDP) measure was used to assess the oral health-related quality of life.* Results.* Four groups of older people with different tooth types were studied. 216 (26.6%) had natural teeth (NT). 189 (23.3%) had natural and replaced teeth (NRT). 167 (20.6%) had below the minimum number of teeth but had no dentures (Edent) and 240 were edentate with complete dentures provided by the Royal Project (ECD) (29.6%). Overall, 36.5% had at least one oral impact. Eating was the most affected oral impact. When compared to the group with natural teeth (NT), the Edent group was significantly more likely to report having impacts on eating OR = 6.5 (3.9–10.9), speaking clearly OR = 43.7 (12.7–15.07), emotional stability OR = 16.5 (6.0–45.6), and social contacts OR = 4.6 (2.2–9.5) (*p* < 0.001).* Conclusion.* Those who are edentulous are much more likely to have an oral impact on their daily performances than those provided dentures. Provision of dentures may lead to improvement of considerable oral impacts.

## 1. Background

In 2005, Thailand reached the aging society whereby ten percent of the total population were sixty years of age or older [1]. This proportion is expected to increase to 20% by 2025 [2]. Consequently, aging has become an important issue for health and oral health care providers. The World Health Organization (WHO) has included oral health as a component of active aging. In the WHO policy framework of active aging, maintaining natural teeth throughout life could promote health [3]. Therefore, having good oral health should contribute to older people's health and quality of life.

Loss of teeth is the most common dental problem among the older people [4–6]. Having missing teeth has a measurable impact on daily living especially in older people [7–10]. Denture status has been related to quality of life [11, 12]. Subjects with missing teeth who did not wear dentures had lower oral health-related quality of life scores than those who wore removable or complete dentures [12]. However, those studies which were performed in Germany included a wide age range from 16 to 79 years old and were not aimed at studying the impact of the provision of dentures on the oral health-related quality of life in an older age Asian group.

The most recent Thai National Oral Health Survey reported that 7.2% of people aged 60 and above were edentulous [13]. To improve inequity in access to oral care and to improve the oral health of the Thai older people, the Thai Government launched the Royal Denture Project to provide free complete dentures or removable dentures to those who needed them. Older people who participated in the Royal Denture Project were provided with dentures by dentists. National guidelines to make the dentures were developed by an expert panel of prosthodontists from Thai dental schools.

There have been a number of studies showing improved oral health quality of life after the provision of dentures. According to other studies, wearing dentures helps rehabilitate the oral health status for the older people, and it improves the chewing ability and makes oral health functions better. This therefore improves their quality of life [14–18]. Ettinger also found that good-quality complete dentures increased chewing ability [19]. The improvement of the quality of life after receiving new dentures was also reported in a study of French older people [20] and a Canadian study [9]; however, no studies have involved Asian samples or places where the dentures were provided by a government.

This study was conducted with the objective of assessing the relationship between dental status and oral health-related quality of life in older Thai people.

## 2. Methods

This cross-sectional study was approved by the Ethical Committee of the Faculty of Dentistry, Chiang Mai University.

## 3. Subjects

Subjects were independently living older persons aged 60 years and above. They were only included in the study if they had adequate general health; that is, they could have a systemic disease but were able to live independently. They were excluded if they had any soft tissue oral lesions, had oral cancer, had taken any medicine which affected salivary secretion, or had any history of an accident to the head and neck area. Participants provided informed consent before inclusion.

## 4. Study Sites

Five cities were purposively selected to represent different parts of Thailand: Chiang Mai for the upper northern region, Phitsanulok for the lower northern region, Songkhla for the southern region, Chaiyaphum for the northeastern region, and Bangkok for the metropolitan area. At each site, approximately 30–40 subjects were recruited for each group. A total of 120 to 180 participants were recruited from each study site.

## 5. Subject Recruitment

The ECD and NRT were purposively sampled from those who had undergone treatment for complete dentures or acrylic partial dentures under the Royal Denture Project. Subjects in ECD group were those who had worn their new dentures for at least one year prior to clinical examination and the interview period. NRT subjects were those who had at least 16 missing teeth and had removable partial dentures made under the Royal Project to replace missing teeth at least one year prior to the study. All remaining teeth of the NRT subjects were functional. The other two groups of subjects (Edent and NT) were recruited from older people who resided in the same community as the ECD and NRT groups and were of comparable economic status as those two groups. The edentates (Edent) were persons who had become edentate for one year or more prior to the study period but had not had dentures made. The NT group subjects were those who had at least 4 occluding pairs of posterior teeth, had no or minimal mobility, and were free from the need for emergency treatment and pain. The NT and Edent groups did not have the history of wearing dentures.

## 6. Clinical Examination

All subjects were clinically examined by trained dentists. Training sessions for clinical assessment were organized to calibrate the examiners. All clinical examinations were done under natural light in community settings. The clinical data assessed for dentate subjects included numbers and status of natural teeth, types of dentures, and occluding posterior pairs of teeth in the premolar and molar areas. Denture-bearing areas in edentate participants were examined for the presence of lesions, condition of dentures, and numbers of occluding posterior pairs.

## 7. Interview and the Assessment of Oral Impacts on Quality of Life

Prior to the assessment for the impact on their quality of life in all subjects, cognitive impairment was assessed using the Mini-Mental State Examination (MMSE) which had been translated into Thai [21, 22]. Only those who scored ≥18 on MMSE were included in the study. The face-to-face interviews were conducted by trained interviewers to collect data on basic sociodemographic variables, such as age, gender, and level of education. Oral health-related quality of life (OHRQoL) data were assessed using the Oral Impacts on Daily Performances (OIDP) indicator. This OHRQoL index has been widely accepted and previously translated and employed in Thai populations [7, 23]. The interview was conducted by a team of interviewers who had previously undergone 2-day training and calibration course on the OIDP measure.

## 8. Calculation of the OIDP Score

The OIDP instrument is a composite OHRQoL measure. It is a theoretically sound, relatively brief instrument that attempts to measure oral impacts which can seriously affect a person's daily life. In this study, we focused on 8 basic daily life activities and behaviors. The OIDP instrument measures both the frequency and the severity of impacts on daily activities affected by the oral conditions in the last 6 months. This OIDP indicator provides scores ranging from 0 to 25 for each daily activity and from 0 to 200 for overall impact on daily performance. In addition to the OIDP scores, the intensity of the oral impacts was calculated. Intensity was defined according to the highest daily performance score among the eight performances, which were classified into five levels: very little, little, moderate, severe, and very severe. The intensity illustrates how severe the impacts of mouth were on daily life [24].

## 9. Statistical Analysis

Descriptive statistics were used to describe the prevalence, the severity, and the intensity of Oral Impacts on Daily Performances among subjects with different dental status. Odds ratio and multivariate logistic regression analysis were used to test significance of the independent variables and the difference in the overall oral impact scores in groups with different dental status. Unadjusted odds ratios were calculated by comparing proportion of subjects who reported suffering in each daily life activity among Edent, ECD, and NRT groups to those in the reference NT group. Then, an adjusted odds ratio was calculated where sex, age, marital status, education level, and systemic disease were added to the calculation. 95% confidence intervals and *p* values were also computed and presented.

## 10. Sample Size

Subjects were categorized into four groups by dental status: edentulous but no denture (Edent), edentulous with complete dentures (ECD), with natural and replaced teeth (NRT), and with only natural teeth (NT). Sample size calculation was aimed at detecting differences of OIDP scores among the 4 study groups. Using the standard deviation of the total OIDP scores from a previous study [7] under conditions of a 95% confidence interval and 85% power of test, the minimum sample size was computed to be 160 for each group.

## 11. Results

All 848 subjects approached consented to participate in the study. Three subjects were excluded as their MMSE scores were ≤18. Thirty-three subjects were below 60 years of age and did not complete the clinical examination or the interview. Only 812 subjects fully participated in the study: 353 (43.5%) males and 459 (56.5%) females. The average age of subjects was 69.8 ± 6.1 years. 216 subjects (26.6%) had natural teeth (NT); 189 (23.3%) had natural and replaced teeth (NRT); 167 (20.6%) were edentate but did not have a denture (Edent); and 240 (29.6%) wore complete dentures (ECD). There were more females than males in all groups.

Table 1 presents the impact of oral health on different daily performances. Among all subjects, approximately a third (36.6%) had at least one OIDP impact which affected their daily performances in the past 6 months. Eating was the most commonly impacted performance (27.1%). The average total OIDP scores for ECD were 1.6 ± 4.5, similar to 1.9 ± 5.0 for NRT and 1.4 ± 4.2 for NT groups, but considerably less than 9.6 ± 13.6 for Edent. Eating was the most frequently affected oral performance in all four groups. The mean total OIDP score for eating was the highest of all oral impacts. The other two most frequently influenced activities were speaking clearly (11.9%) and emotional stability (9.2%). Doing light activities was the performance least affected by oral status (1.2%).

The subjects in four different dental status groups reported significant differences in oral impacts on eating, speaking clearly, cleaning mouth, smiling, emotional stability, and social contacts (*p* < 0.05). The Edent group, compared to the other three groups, reported the highest proportion of oral impacts on all eight performances, significantly so with respect to eating, speaking clearly, cleaning mouth, smiling, emotional stability, and social contact (Table 1).

In the analysis for severity and intensity of the oral impacts, among those who reported having any impacts, 31.7% reported impacts of severe or very severe intensity; 40.1% reported that their impacts were of only little or very little intensity (Table 2). When analyzing the impact on each performance separately, the top three impacts reported to be severely affected were eating (35.5%), emotion (28.4%), and smiling (28.0%). Doing light activities was the least severely affected (20.0%). The top three impacts reported to be very severely affected were social contact (24.5%), smiling (20.0%), and eating (18.2%).

Table 3 presents the results of a multivariable logistic regression analysis. The odds ratios of Edent, ECD, and NRT reporting having oral impacts on each performance were compared to the NT group in both unadjusted and adjusted models. In both the unadjusted and adjusted models, the overall impact and specific significant oral impacts on eating, speaking clearly, emotional stability, and social contacts were 6.5 (3.9–10.9), 43.7 (12.7–15.1), 16.5 (6.0–45.6), and 4.6 (2.2–9.5) times more in Edent subjects compared to their counterparts in the NT group. The ECD and NRT subjects were only significantly more likely to report impacts on speaking than the NT group (OR = 5.4 (1.6–18.9) and 7.5 (2.2–25.6)).

## 12. Discussion

More than one-third of the subjects with all types of dental status (36.5%) reported that they had experienced one or more oral impacts which affected their daily life. As the samples from this study were not representative of the whole population of Thai older people, it is not appropriate to directly compare the present findings to other studies. However, it is worth noting that the range of oral impacts using the same OHRQoL index for assessment was lower than in other studies, which ranged from 39.1% to 62.9% [25–27] although the proportion of subjects who reported severe or very severe oral impacts was similar to that reported in a group of Northern Thai older people (39.1%) [7]. The strong Buddhist belief may be one of the reasons to explain the lower expression of impacts from oral conditions in our sample. In Thailand, 94.6% of the population are Buddhist [28]. Thais believe that aging is the transiency of life. Under this belief, they may accept the changes and impacts from health condition as part of normal aging phenomena. Losing teeth and changes due to an oral condition are accepted as normal. Therefore, any problems from oral conditions may not be seen as having much effect on their lives as in other populations.

The intensity analysis illustrates how severe the impacts of oral symptoms were on their daily lives. Although the prevalence of the oral impacts was relatively low, many reported their impacts as being from severe to very severe. This finding suggests a strong need to intervene to address oral health issues of the older people, since they may be unlikely to seek treatment for something they believe is normal.

When comparing the oral impacts among older people with different dental status, the group with the highest proportion to report having oral impacts was the edentate group. Among the ECD group, only speaking differed significantly from NT group. These results are consistent with other studies reporting that wearing dentures improves quality of life [14–18]. Ettinger also reported that good-quality complete dentures increased chewing ability [19]. The improvement of the quality of life after receiving new dentures was also reported in a study of French older people [20] and a Canadian study [14]. This suggests that the provision of dentures to older Thai people greatly improves their oral health quality of life, almost to the level of those with adequate natural teeth. In addition, dental status not only can affect the ability to eat but also affects nutrient intake and hence can compromise overall health and well-being [29–31].

Although this study provides evidence for the benefit of providing dentures to the edentulous population as done in the Royal Denture Project, it also provides evidence for the importance of maintaining teeth throughout life. Providing dentures after tooth loss consumes the dentist's time and is costly. In Thailand, the aging population is projected to increase dramatically [32]. The dental manpower will not be able to meet the demand for oral rehabilitation in the future. Therefore, oral health promotion to promote proper oral health care at an earlier stage should be the high priority for the dental public health personnel.

## 13. Limitation of the Study

The cross-sectional design in this study could not allow us to make a direct conclusion that providing dentures to those who were in need could improve their quality of life. While our findings determine a relationship between being provided with dentures and having a higher quality of life, more research needs to be carried out on a longitudinal basis.

## 14. Conclusions

The results suggest that among older Thai people those provided with dentures had important impacts on their oral health quality of life, which provides support for the government effort to provide dentures.

## Figures and Tables

**Table 1:** Comparisons of prevalence and total mean OIDP score of oral impacts on daily performances between the groups.

Oral impacts	Total prevalence	Subjects with different dental status				Total mean OIDP score	*p* value^*∗*^
		Edentate (Edent)	Complete denture (ECD)	Natural and replaced teeth (NRT)	Natural teeth (NT)		
	*n* (%)	*n* (%)	*n* (%)	*n* (%)	*n* (%)	Mean (±SD)	
Overall impacts	297 (36.6)	114 (68.3)	68 (28.3)	60 (31.7)	55 (25.5)		

*Physical performances*							
Eating	220 (27.1)	94 (56.3)	48 (20.0)	41 (21.7)	37 (17.2)	2.8 (±6.1)	<0.001
Speaking clearly	97 (11.9)	59 (35.3)	17 (7.1)	18 (9.5)	3 (1.4)	1.0 (±3.9)	<0.001
Cleaning mouth	37 (4.6)	14 (8.4)	8 (3.3)	4 (2.1)	11 (5.1)	0.4 (±2.4)	0.027
Doing light activities	10 (1.2)	4 (2.4)	3 (1.3)	1 (0.5)	2 (0.9)	0.1 (±1.1)	0.425

*Psychological performances*							
Sleeping and relaxing	49 (6.0)	12 (7.2)	10 (4.2)	14 (7.4)	13 (6.0)	0.4 (±2.3)	0.403
Smiling	25 (3.1)	23 (13.8)	1 (0.4)	1 (0.5)	0 (0.0)	0.3 (±2.3)	<0.001
Emotional stability	74 (9.1)	35 (21.0)	10 (4.2)	16 (8.5)	13 (6.0)	0.8 (±3.2)	<0.001

*Social performances*							
Social contact	49 (6.0)	40 (24.0)	1 (0.4)	3 (1.6)	5 (2.3)	0.6 (±3.1)	<0.001
*Total mean OIDP score (mean ± SD)*		9.6 (±13.6)	1.6 (±4.5)	1.9 (±5.0)	1.4 (±4.2)	3.3 (±8.0)	

^*∗*^ *p* < 0.05, Chi-square.

**Table 2:** Intensity of oral impacts among 812 Thai older people.

Oral impacts	Subjects with impacts	Intensity				
		*n* (%)				
		Very little	Little	Moderate	Severe	Very severe
	*n* (%)					
Overall impacts	297 (100)	59 (19.7)	60 (20.2)	84 (28.3)	41 (13.8)	53 (17.8)
*Physical performances*						
Eating	220 (100)	32 (14.5)	43 (19.5)	67 (30.5)	38 (17.3)	40 (18.2)
Speaking clearly	97 (100)	26 (26.8)	18 (18.6)	31 (32.0)	6 (6.2)	16 (16.5)
Cleaning mouth	37 (100)	7 (18.9)	8 (21.6)	12 (32.4)	6 (16.2)	4 (10.8)
Doing light activities	10 (100)	2 (20.0)	0 (0.0)	6 (60.0)	1 (10.0)	1 (10.0)
*Psychological performances*						
Sleeping and relaxing	49 (100)	15 (30.6)	11 (22.4)	13 (26.5)	6 (12.2)	4 (8.2)
Smiling	25 (100)	3 (12.0)	3 (12.0)	12 (48.0)	2 (8.0)	5 (20.0)
Emotional stability	74 (100)	14 (18.9)	17 (23.0)	22 (29.7)	13 (17.6)	8 (10.8)
*Social performances*						
Social contact	49 (100)	9 (18.4)	8 (16.3)	19 (38.8)	1 (2.0)	12 (24.5)

**Table 3:** Unadjusted and adjusted odds ratio (95% confidence interval) of overall oral impacts in groups with different dental status.

	Unadjusted	*p* value	Adjusted^*∗*^	*p* value
	odds ratio		odds ratio	
Overall impacts^*∗∗*^				
Edent	6.6 (4.1–10.3)	<0.001	8.1 (4.8–13.5)	<0.001
ECD	1.2 (0.8–1.7)	0.492	1.2 (0.8–1.8)	0.475
NRT	1.4 (0.9–2.1)	0.171	1.3 (0.9–2.0)	0.201
*Physical performances*				
Eating				
Edent	6.4 (4.0–10.2)	<0.001	6.5 (3.9–10.9)	<0.001
ECD	1.2 (0.7–1.9)	0.432	1.1 (0.7–1.8)	0.720
NRT	1.3 (0.8–2.2)	0.246	1.2 (0.7–2.0)	0.507
Speaking clearly				
Edent	38.8 (11.9–126.6)	<0.001	43.7 (12.7–15.07)	<0.001
ECD	5.4 (1.6–18.7)	0.008	4.9 (1.4–17.7)	0.008
NRT	7.5 (2.2–25.8)	0.001	7.7 (2.2–27.2)	<0.001
Cleaning mouth				
Edent	1.7 (0.7–3.8)	0.205	2.3 (0.9–5.7)	0.205
ECD	6.4 (0.2–1.6)	0.346	0.6 (0.2–1.5)	0.346
NRT	0.4 (0.1–1.3)	0.123	0.4 (0.1–1.3)	0.14
Doing light activities				
Edent	2.6 (0.5–14.5)	0.268	3.9 (0.6–27.0)	0.268
ECD	1.3 (0.2–8.1)	0.741	1.2 (0.2–8.7)	0.864
NRT	0.6 (0.5–6.3)	0.646	0.7 (0.6–8.5)	0.788
*Psychological performances* ^*∗∗∗*^				
Sleeping and relaxing				
Edent	1.3 (0.6–2.9)	0.497	1.4 (0.6–3.4)	0.412
ECD	0.4 (0.3–1.6)	0.369	0.7 (0.3–1.8)	0.369
NRT	1.2 (0.6–2.7)	0.577	1.3 (0.6–2.9)	0.529
Emotion stability				
Edent	13.4 (5.1–34.8)	<0.001	16.5 (6.0–45.6)	<0.001
ECD	0.2 (0.0–1.5)	0.115	0.2 (0.0–1.9)	0.148
NRT	0.7 (0.2–2.9)	0.602	0.8 (0.2–3.5)	0.797
*Social performances*				
Social contact				
Edent	4.3 (2.2–8.5)	<0.001	4.6 (2.2–9.5)	<0.001
ECD	0.7 (0.3–1.6)	0.369	0.7 (0.3–1.8)	0.506
NRT	1.4 (0.7–3.1)	0.343	1.2 (0.6–2.8)	0.551

^*∗*^Adjusted with sex, age, marital status, education level, income, and systemic disease.

^*∗∗*^Reference group: natural teeth group.

^*∗∗∗*^As no subjects in a reference group reported impact on smiling, the odds ratios were not calculated in this performance.
